# Molecular structural dataset of lignin macromolecule elucidating experimental structural compositions

**DOI:** 10.1038/s41597-022-01709-4

**Published:** 2022-10-22

**Authors:** Sudha cheranma devi Eswaran, Senthil Subramaniam, Udishnu Sanyal, Robert Rallo, Xiao Zhang

**Affiliations:** 1 Bioproducts Sciences and Engineering Laboratory, Washington State University, 2710 Crimson Way, Richland, WA 99354 USA; 2 Voiland School of Chemical Engineering and Bioengineering, Washington State University, Richland, WA 99354 USA; 3 Pacific Northwest National Laboratory, 902 Battelle Blvd, Richland, WA 99354 USA

**Keywords:** Supramolecular polymers, Scientific data

## Abstract

Lignin is one of the most abundant biopolymers in nature and has great potential to be transformed into high-value chemicals. However, the limited availability of molecular structure data hinders its potential industrial applications. Herein, we present the Lignin Structural (LGS) Dataset that includes the molecular structure of milled wood lignin focusing on two major monomeric units (coniferyl and syringyl), and the six most common interunit linkages (phenylpropane β-aryl ether, resinol, phenylcoumaran, biphenyl, dibenzodioxocin, and diaryl ether). The dataset constitutes a unique resource that covers a part of lignin’s chemical space characterized by polymer chains with lengths in the range of 3 to 25 monomer units. Structural data were generated using a sequence-controlled polymer generation approach that was calibrated to match experimental lignin properties. The LGS dataset includes 60 K newly generated lignin structures that match with high accuracy (~90%) the experimentally determined structural compositions available in the literature. The LGS dataset is a valuable resource to advance lignin chemistry research, including computational simulation approaches and predictive modelling.

## Abstract (summary)

**Table:** 

Measurement(s)	molecular structure
Technology Type(s)	Computer Modeling
Factor Type(s)	monomer ratio • bond frequency • degree of polymerization
Sample Characteristic - Organism	coniferous (softwood) • deciduous (hardwood)

## Background & Summary

Lignin is one of the most abundant biopolymers in nature followed by cellulose. Natural biopolymers such as DNA contain well-defined arrangements of monomers and linkages that can be consistently determined through experimental characterization. In contrast, lignin’s monomer sequence and structure are not well-defined and present extremely complex chemical structures resulting from an irregular biosynthesis process^1,2^. Lignin is not used to its fullest potential due to uncertainties in the characterization of its primary molecular structure.

Lignin structures are formed primarily by three main repeating units (monolignols), arranged in random sequences. The major contributors to lignin’s primary structure are p-coumaryl (H), coniferyl (G), and sinapyl (S) alcohols^3,4^. The monomers radically couple and cross-link combinatorially^5^ to form multifaceted lignin structures. During lignin biosynthesis (or lignification), monomers are transported to the cell wall where they are polymerized in a combinatorial fashion by free-radical coupling mechanisms in a reaction mediated by peroxidases, generating a variety of structures within the lignin polymer^3,6,7^. Lignin’s structural composition varies between plant species and changes depending on the tissue, cell location or environmental conditions^8–10^. In addition, major structural changes occur during the lignin separation process^11,12^. During the last decades, various applications of lignin have been investigated, either as a macromolecule or after depolymerization into lower molecular weight compounds. Although there are numerous studies on lignin polymerization process and depolymerization^13^, there is still a lack of curated lignin structure datasets that can support lignin chemistry research.

Molecular structure and the length of monomer chains determine polymer properties (e.g., strength, solubility, heat resistance). Since there may be multiple molecular structures consistent with a given average chemical composition, elucidating the correct molecular structure and structural variations responsible of specific property profiles is a key challenge in lignin chemistry. Current advances in polymer science and computational modelling^14^ provide new insight on the structural features of this macromolecule and guide bioengineering strategies for achieving structures with targeted properties. Experimental techniques such as spectroscopy (e.g., NMR and FT-IR) or wet chemistry (e.g., thioacidolysis, nitrobenzene oxidation, and hydrogenolysis) are key to reveal the molecular features of lignin^1,15,16^ and provide statistical descriptions of the polymer structure. Alternatively, computational methods can be used to predict lignin structure and properties. Multiple research efforts have modelled the lignification process using a variety of simulation techniques parameterized by experimental data on monomer and bond distributions^17–21^. Simulation studies on lignin structure formation are often carried out by adding monomeric units to reactive sites in the polymer^17–19,22^.

This paper describes a novel lignin polymer dataset, herein referred as lignin structural dataset (LGS), containing computer-generated molecular structures of milled wood lignin (MWL). The LGS dataset includes structural isomers for experimental data on spruce (softwood) and birch (hardwood) MWL. This resource provides data for 60 K lignin molecules (6.3 K softwood structures and 53.7 K hardwood structures) with varying degree of polymerization (DP) in the range of 3 to 25. The LGS dataset, available on Figshare^23^, can be readily used in studies aimed at understanding lignin’s structural properties using parameterized force fields^24^ and molecular dynamics approaches^21,25^. The structural information contained in the dataset provides a unique resource for simulating the depolymerization of lignin into lower molecular weight compounds. In addition to the structure data, the paper also describes a new computational approach to sequence-controlled polymer generation. Sequence-controlled polymers are macromolecules in which monomer units of different chemical nature are arranged in an orderly fashion^26^. A polymer formed by radical chain‐growth polymerization is also considered as a sequence‐controlled polymer, although it is also a nonuniform polymer with chains of different lengths and slightly different composition^27^. The tool can generate chemically correct and legible 2D structures of the MWL for hardwood, softwood and herbaceous wood types. The code used to generate and validate lignin structures is also provided for reproducibility.

## Methods

The computational approach used to generate the LGS dataset is based on sequence-controlled polymer growth parameterized to match experimental lignin structure data. Lignin’s structural heterogeneity can be characterized by the inter-unit linkages and functional groups attached to phenyl propane units. Inter-unit linkages are present in two major categories corresponding to ether bond linkages (e.g., β-O-4, α- O-4, 4-O-5) and carbon-carbon linkages (e.g., β–β, β-5, 5–5). The relative abundance of each linkage in native lignin varies from plant to plant with the β-O-4 linkage being the most abundant^28,29^. The major functional groups present in lignin structures include hydroxyl, methoxy, carbonyl and carboxylic groups. The proportion of these groups in phenyl propane units depends on the species genetic origin and isolation processes.

Lignin structure compositions were analyzed from experimental studies on MWL of different wood species. Table 1 summarizes experimentally determined frequencies of primary monolignols and linkages in softwood and hardwood species.

**Table 1:** Primary monolignol and main linkage frequency in hardwood and softwood plant types.

	Coniferous or Softwood %	Deciduous or Hardwood %	References
**Primary Monolignols**			
G: Coniferyl alcohol	>95	25–50	^2^
S: Sinapyl alcohol	0–1	45–75	^2,60^
H: p-Coumaryl alcohol	<5	0–8	^2^
**Major Linkages**			
Phenylpropane β-aryl ether (β-O-4)	45–50	50–65	^61,62^
Resinol (β-β′/γ-O-α/α-O-γ)	2–6	3–16	^61,62^
Phenylcoumaran (β-5∕α-O-4′)	9–12	3–11	^61,62^
Biphenyl (5-5′) and Dibenzodioxocin (5-5′/β”-O-4/α”-O-4′)	2.5–11	<1–4	^12,62,63^
Diaryl ether (4-O-5)	2, 4–8	2, 7	^61,62^

Experimental characterization^3,5,10,30–32^ distinguishes two main classes of lignin biopolymers, G type lignin which is typical of conifers or softwood, and SG type lignin which is formed in deciduous plants or hardwood. The presence of H monomers is minimal in both of these lignin types^33^. The main structural variations of lignin include straight chain (linear) structures, branching structures, and cross linking with carbohydrates^34^. Two forms of coupling reactions could occur during lignin biosynthesis. First, linear reactions which contribute to extending the macromolecule by coupling new monomer units with β-O-4, β–β and β-5 linkages to form monomer-monomer and oligomer-monomer couplings. Second, branching reactions that form oligomer-oligomer couplings with 5-5 and 4-O-5 linkages^3,26,30,35^. It is important to note that units with 5-5 linkage have an alternate form with an 8-member ring structure named dibenzodioxocin (DBDO)^36,37^ which is predominantly present in softwood lignin. Spirodienone (β-1′/α-O-α′) has also been reported as a possible linkage with low prevalence (~3–4 in 100 phenylpropanoid units)^3,38^.

### Generation of lignin structures

**Fig. 1:**
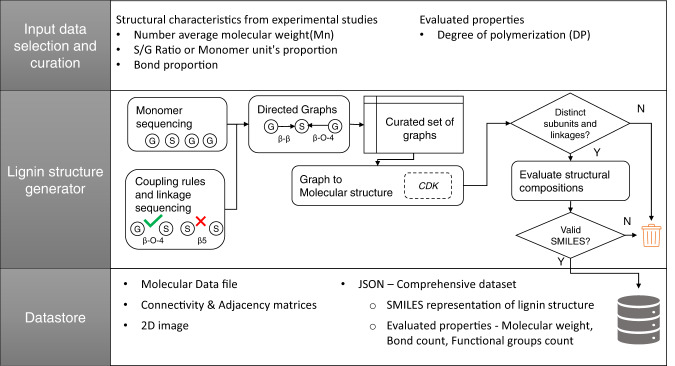
Workflow for generation and validation of lignin structures included in the LGS dataset.

The structure generation approach used to create the LGS dataset doesn’t assume that lignin conforms to a specific sequence of monomers and bond types. Instead, our work assumes that lignin structures are created as the result of a random or near-random coupling of primary monomers, where each monomer can couple in any of all possible ways. To capture lignin’s structural variability, we have developed a generative model based on a combinatorial approach that uses a sequence-controlled method to create theoretical lignin structures. The algorithm starts by defining the primary monomer units followed by the application of molecular connectivity rules based on experimental studies^3,5,10,30^ to generate plausible monomer sequences. Subsequent steps generate all possible molecular arrangements with similar structural features (i.e., monomer ratio and linkages). The validity of the resulting lignin polymers is then corroborated by comparing their features (e.g., functional groups, linkages, and end groups) with experimental data as well as by matching with molecular fingerprints of structures extracted from recent studies^20,21^ on lignin structure simulation. Figure 1 summarizes the workflow used for structure generation with details on the required input information and the structure of the generated LGS dataset.

The input information needed to execute the workflow includes the S/G ratio, bond ratio, and degree of polymerization. Experimental characterization data used to bootstrap the structure generation were obtained from analytical results reported in previous studies^5,30,39,40^ on softwood and hardwood lignin samples. The degree of polymerization (DP) is computed as the ratio between the weight average molecular weight (M_w_) of lignin polymer and the weight average molecular weight of phenylpropane monomer (M_0_) (i.e., DP = M_w_/M_0_)^41^. The degree of polymerization represents the number of monomer units in each lignin structure. The structure generation workflow starts with sequencing lignin monomers and the generation of linkages, followed by the creation of topological matrices and molecular graph representations. In subsequent steps, graphs are converted to canonical molecular structures and the consistency of the generated structures is verified by comparison with experimental observations and simulated data. Finally, all validated structures are assembled to create the LGS dataset. The next subsections provide specific details on each workflow step.

### Sequencing lignin monomers and conditional linkage generation

**Fig. 2:**
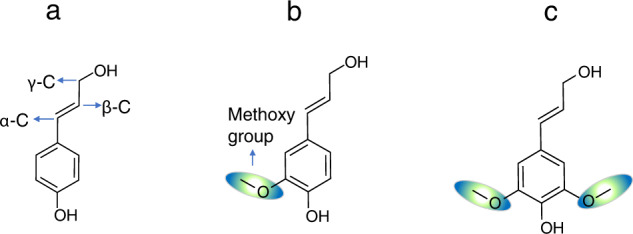
Primary monomer units used for lignin sequence generation. (**a**) base unit corresponding to p-coumaryl alcohol or H monomer, (**b**) Coniferyl alcohol or G monomer and (**c**) Syringyl alcohol or S monomer.

Phenyl propane units (Fig. 2) form the core backbone of the lignin structure^42^. The base unit in the backbone contains structural features of p-coumaryl alcohol (H), in which an allylic alcohol moiety is connected to the para position of phenolic ring. The three carbon atoms present in the aliphatic side chain are located at α, β and γ positions respectively. During lignin structure generation, the H monomer is considered as the base unit whereas coniferyl alcohol (G monomer) and syringyl alcohol (S monomer) are modifications of the base unit with additions of one (position 3) and two (position 3 and 5) methoxy groups, respectively.

The lignin structure generator uses a permutation approach to create lignin structures including all possible combinations of H, G, and S monomers. However, since most available experimental data corresponds to G and SG type lignin structures, only S and G monomers were considered for inclusion in the LGS dataset. Table 2 summarizes the linkage patterns used to generate lignin structures. The sequence generation process creates all possible arrangements of the primary monomer units to produce a lignin chain. For instance, in a G type structure with five monomers, the repeating unit will be [G, G, G, G, G] with a single monomer sequence whose length is controlled by DP. For SG type structures, a set of permutations based on the S/G ratio and DP is used to generate all possible monomer arrangements.

**Table 2:** Linkage patterns used for structure generation.

Linkages	Linkage types	Structure	Dimer structures	Structure Type
Carbon-oxygen bonds (C-O-C)	β-O-4		Phenylpropane β-aryl ether	Linear
	4-O-5		Diaryl ether	Branched
Carbon-carbon bonds (C-C)	5-5		Biphenyl	Branched
Carbon–oxygen & Carbon–carbon bonds	β-5/α-O-4		Phenylcoumaran	Linear
	β-β/γ-O-α/α-O-γ		Resinol	Linear
	5-5/β-O-4/α-O-4		Dibenzodioxocin	Branched

**Fig. 3:**
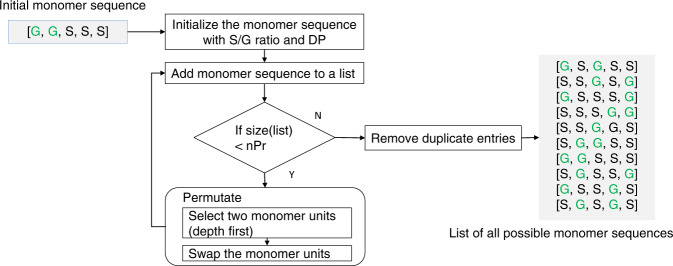
Workflow description of the recursive permutation algorithm and an example of monomer sequence generation for 5 monomer units with S/G ratio of 1.8.

The sequence generation code implements Heap’s permutation algorithm^43^ using a recursive function adjusted to generate all possible permutations of *n* monomer units (Fig. 3). Although there are ^n^P_r_ possible monomer permutations, filtering duplicate arrangements reduces the number of candidate structures. For instance, running the sequence generation algorithm with 5 monomer units generates 10 unique sequences for a S/G ratio of 1.8 as illustrated in Fig. 3.

The linkage sequence is generated using a similar approach, except that the bonding position is rearranged based on monomer labels (i.e., G, S) and possible linkages between monomers. Table 3 presents the coupling patterns for monomers and oligomers reported in the literature^3,5,30,39^. Structures are linearly coupled when one of the favored sites is the β position. Coupling reactions between oligomers produce 5-5 and 4-O-5 branched structures.

**Table 3:** Monomer coupling, and cross-coupling patterns extracted from literature. *M1, M2 represent the individual monomer unit and O1* and *O2 represent two or more units connected* forming *a lignin chain* (L).

Linear									Branched			
Dimer structures					Oligomer and monomer				Oligomer cross-coupling			
M1	M2	β-O-4	β-β	β-5	O1	M1	β-O-4	β-5	O1	O2	4-O-5	5-5
G	G	✓	✓	✓	L-G	G	✓	✓	L-G	L-G	✓	✓
S	S	✓	✓	✗	L-S	S	✓	✗	L-S	L-S	✗	✗
S	G	✓	✓	✓	L-S	G	✓	✓	L-G	L-S	✗	✗
G	S	✓	✓	✗	L-G	S	✓	✗	L-S	L-G	✓	✗

Experimental analysis data from previous studies^5,30,44,45^ have shown different branching structure patterns associated with 5-5 and 4-O-5 linkages in native/MWL lignin. The different branching patterns considered in this model are phenolic 5-5^45^, phenolic 4-O-5^5,30,45^, phenolic dibenzodioxocin (DBDO)^5,45^, non-phenolic DBDO^30,44,45^ and etherified 5-5^45^.

### Generation of topological matrices and molecular graph representations

**Fig. 4:**
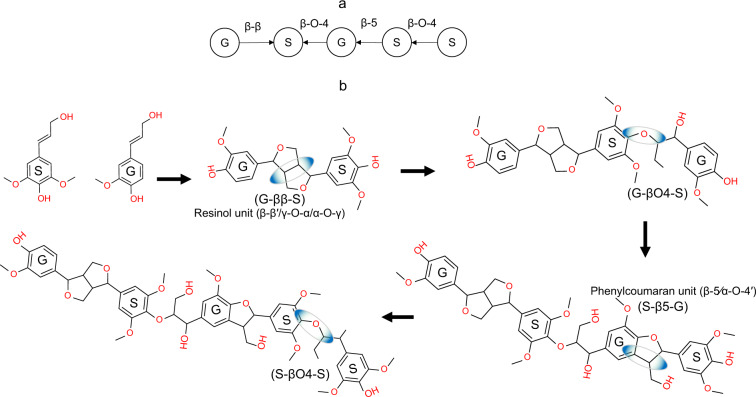
Representation of linear chain structures. (**a**) Graph-based encoding of a linear chain, (**b**) 2D representation of a linear lignin chain structure formation.

**Fig. 5:**
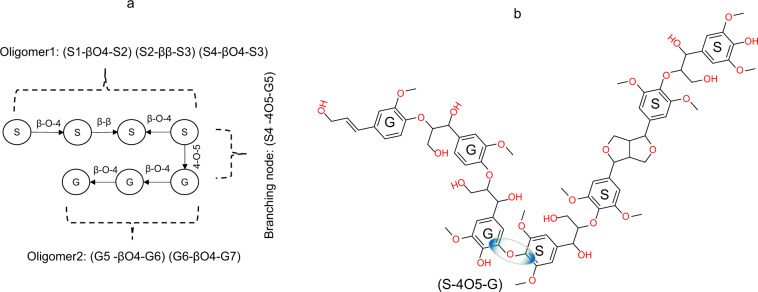
Representation of branched chain structures. (**a**) Graph-based encoding of a branched structure encompassing two oligomers and a branching node, (**b**) 2D representation of the lignin branched structure.

The structure generation mimics the monomer sequencing in chain-growth polymerization by assigning probabilities to the presence of specific monomer pairs. Resulting molecular structures can be represented as graphs where nodes represent monomer units, and the edges correspond to covalent bonds formed during the polymerization process. For example, in case of outgoing edges for linkage type β-O-4, the β position of the monomer represented in the first node is connected to the O-atom attached to the 4^th^ carbon in the ring structure of the monomer in the second node. Studies on lignin structure report that monomers are bidirectionally linked^5^. To account for link directionality, labels are stored as tuples of variables (e.g., (G, S) (S, G) (G, S) (S, G)) that represent parent-child relationship with bonds represented as directed edges denoting the linkage direction. Monomer sequences and bond patterns generated in the previous step are processed to create linear and branched structures. Linear chains are created by adding monomers one-by-one to the polymer (i.e., endwise lignin growth). Branching chains are formed by coupling fragmented linear chains (i.e., two lignin oligomers) using the coupling patterns described in Table 3. Figures 4 and 5 illustrate the development of linear and branched structures.

**Fig. 6:**
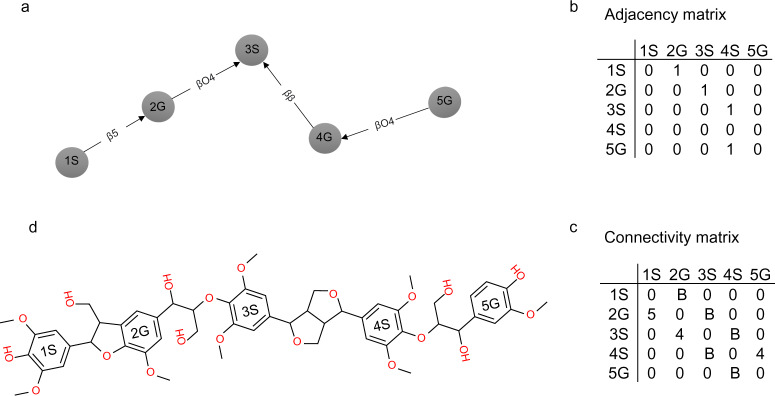
Adjacency and connectivity matrices (**a**) Directed graph, (**b**) Adjacency matrix, (**c**) Connectivity matrix, (**d**) Molecular graph.

Molecular graphs can be efficiently represented in tabular form as a pair of topological matrices^46^ that define the relationship between monomer units. The information in the topological matrices includes linkages (i.e., adjacency matrix) and bond types (i.e., connectivity matrix). These matrices identify the bond direction and occupied bond position for each graph node (e.g., β carbon as ‘B’, 5th carbon for G unit as ‘5’, 4-O position in the ring as ‘4’). Figure 6 shows the directed graph and 2D molecular representations of a lignin polymer together with the structure encoding using topological matrices. Additional information describing the format of the topological matrices can be found in the Supporting Information (Figure F).

### Generation of molecular structures and consistency validation

The molecular structure of a lignin is represented using the canonical SMILES (Simplified Molecular Input Line Entry System) notation which represents the molecular structure in a string format^47^. Directed graphs are converted to canonical SMILES by generating the monomer molecular graph and mapping the respective linkages using the Chemistry Development Toolkit (CDK)^48^, a widely used open-source cheminformatics toolkit. Details on the software architecture used to implement the LGS generation tool are provided in the supporting Information (Figures A and B). The LGS tool allows the user to generate different structural variations for a given set of experimental observations by configuring the required parameters such as monomer ratio (S, G and H), bond frequencies (β-O-4, β–β, β-5, 4-O-5, 5-5 and DBDO) in the project configuration file (project-config.yaml). Finally, generated structures are checked for the acceptance criteria that include structures with unique and valid SMILES representation; and number of nodes matching the requested degree of polymerization. A complete dataset description and structure validations from experimental results are provided in the following sections.

## Data Records

**Fig. 7:**
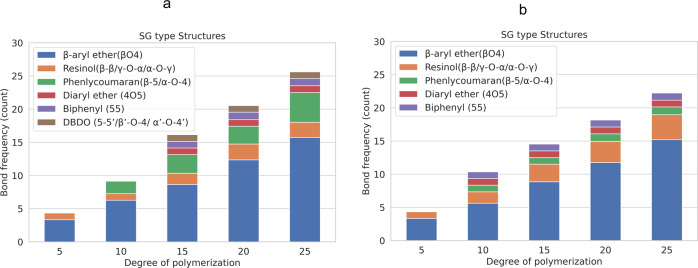
Summary of bond-type frequencies with respect to DP in generated structures from LGS dataset (**a**) G type structures and (**b**) SG Type structures.

The LGS dataset provides structural information for 60 K unique lignin polymers including G and SG type structures with degree of polymerization in the range of 3 to 25. The LGS dataset can be accessed from Figshare^23^. The subset of spruce MWL structures (G type) contains 6.3 K polymers whereas the remaining 53.7 K polymers correspond to birch MWL structures (SG type). Evaluated features of softwood and hardwood MWL structures from the dataset align with structural features reported in experimental studies on lignin structural composition (Table 1). Figure 7 shows the linkage counts for the generated structures. The β-O-4 linkage is predominant across both types of wood with percentages in the range of 50-65%, which agrees with the experimental data summarized in Table 1. For G type structures, the β5/55/DBDO linkages are predominant (up to 6%) whereas 4-O-5 linkages are more frequent in SG type structures (1-6%).

**Fig. 8:**
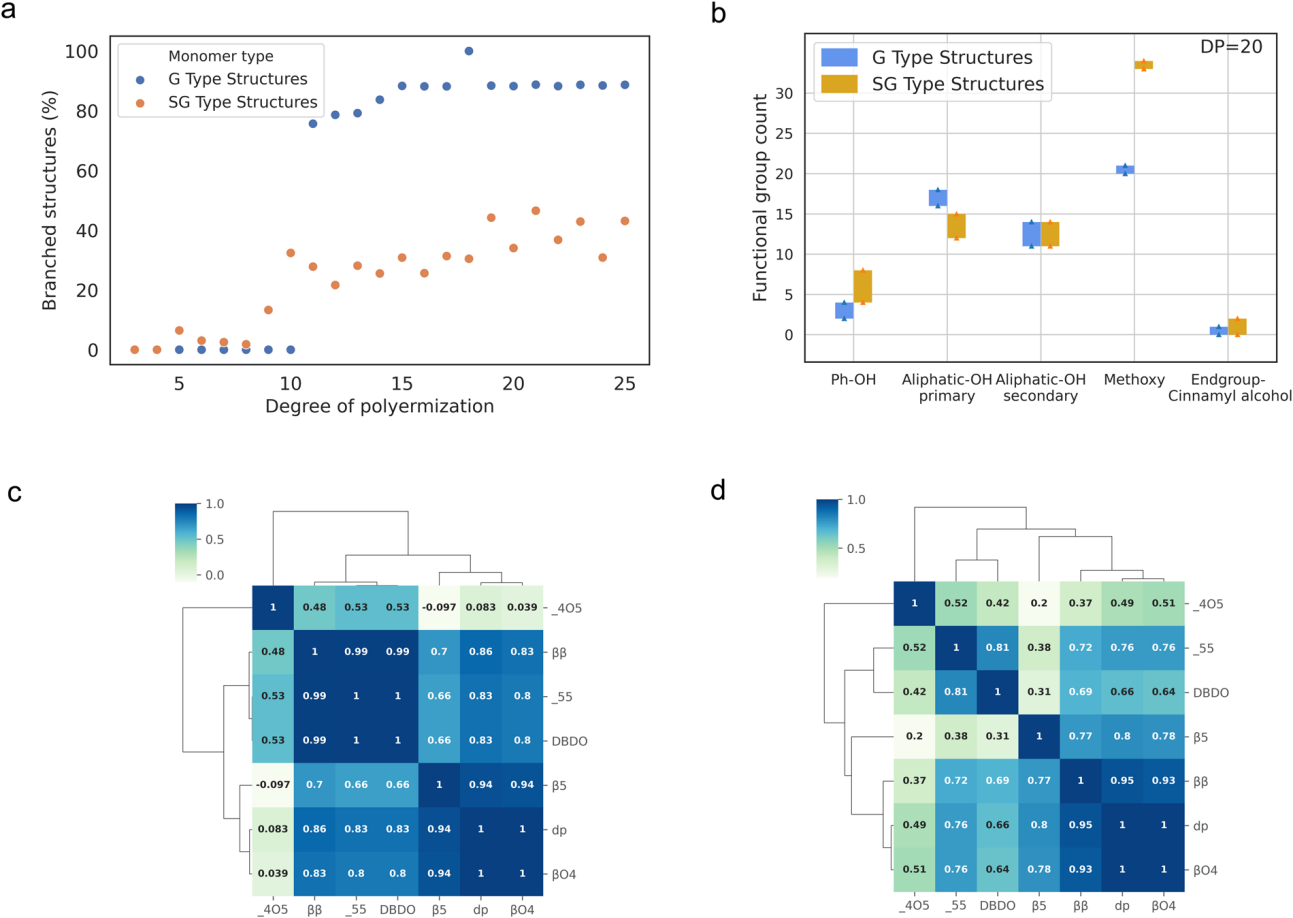
Structural features in the LGS dataset (**a**) Percentage of branching structures, (**b**) Functional group counts for structures with a degree of polymerization of 20, (**c**,**d**) Clustered heatmap of the correlation of bond frequencies and DP in the data subsets corresponding to G and SG type lignin, respectively.

Figure 8 summarizes the main structural features of the macromolecules included in the LGS dataset. Figure 8(a) depicts the ratio of branched structures present in relation to DP in the dataset. Linear chains are the predominant structures for DP up to 4 for both G type and SG type structures. In G type structures, the degree of branching increases abruptly up to 85% when DP is greater than 10. In contrast, for SG structures increasing DP results in branching degrees in the range of 15–30%. The abrupt transition observed for G type structures is due to the presence of vacant 5^th^ carbon in the ring which enables the possibility of forming 5-5 or 4-O-5 structures with increased number of monomer units.

The analysis of functional group counts reveals further structural variations across G and SG type structures. Figure 8(b) depicts the variation in functional group counts for G and SG structures with a degree of polymerization of 20. Since β-O-4 is the major linkage present in both structure types, phenolic hydroxyl groups are mostly condensed. The higher frequency of resinol (β-β/γ-O-α/α-O-γ) structures reduces the number of free primary aliphatic hydroxyl groups in SG type structures. The presence of methoxy groups in SG structures is higher than for G structures. These observations align with previous studies on lignin’s structural charateristics^12^.

Figure 8(c,d) visualize the association of structural features (i.e., linkage and DP) across G and SG type structures. The analysis of the clustered heatmaps reveals structural commonalities and variation patterns. The β-O-4 linkage is strongly correlated with DP in both structure types. In G type structures, β-β bonds have higher correlation with 5-5 and DBDO linkages. The number of β-β bonds restricts the chain growth linearly and generates shorter oligomers which can then couple by 5-5 and 4-O-5 linkages to form branched structures. For SG type structures, the 4-O-5 linkage has higher correlation with DP relative to G structures. This is due to the presence of a methoxy group in the ring structure of syringyl alcohol that limits the formation of 5-5 or DBDO structures in favor of 4-O-5 structures.

### File format

The LGS dataset provides lignin structures in three different formats to facilitate the compatibility with a variety of computational chemistry software platforms (e.g., ADF^49^, Avogadro^50^) and data analytics tools:

- Application specific files: Molecular data files (MOL), which is a widely used chemical structure file format supported by most software packages for molecular dynamics. The data file contains 3D conformation of the generated molecule, which can be used in molecular editors such as Avogadro to visualize and analyze the spatial arrangements.
- Tabular files: Text files using the Comma Separated Value (CSV) format that represent each molecular graph using connectivity and adjacency matrices.
- Key/value file: Text files using the JavaScript Object Notation (JSON^51^) format that provide comprehensive information that includes the definition of molecular structures using SMILES together with properties such as molecular weight, functional group counts and bond ratios. This file also serves as catalog of the structural information with respect to specific DP such as S/G ratio, bond frequencies, SMILES string, etc., Lignin id (lg_id) in the JSON object provides a unique identifier to locate the properties of specific structures in *.mol and *.csv files, respectively.

Detailed file structure definitions and examples for each of the three data formats are provided in the Supporting Information (Figures E, F and G).

### Visualization of the LGS dataset

**Fig. 9:**
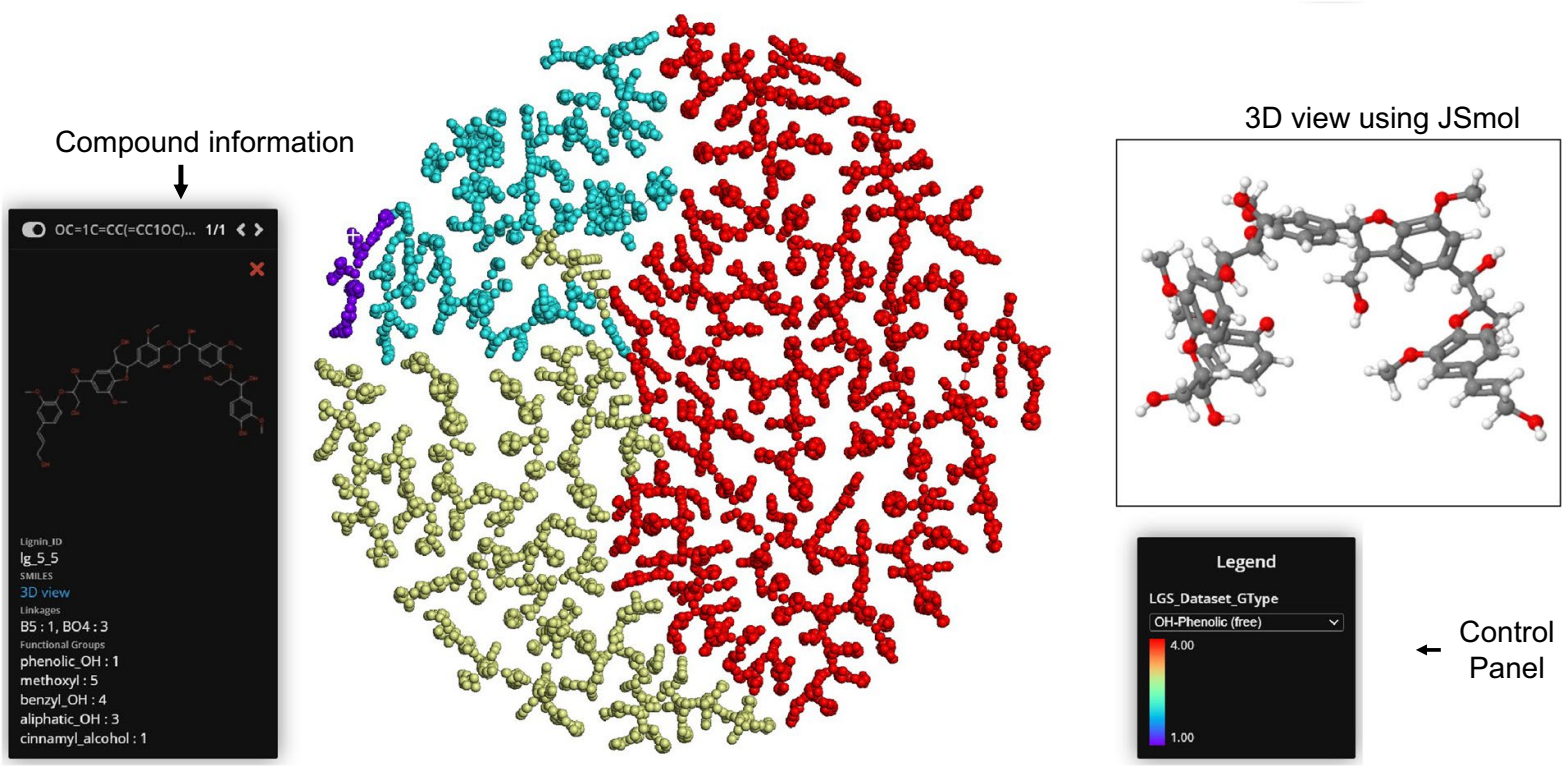
Sample TMAP visualization for G type structures. The color coding is based on the number of free phenolic-OH groups in the lignin molecule. Clicking on an individual datapoint in the tree-view, displays detailed information including structural features and a link to the 3D view.

Figure 9 depicts the Tree MAP (TMAP) projection of calculated MinHash fingerprints (MHFP6)^52^ for a subset of G type lignin structures. The algorithm is an interactive data visualization method that maps high-dimensional chemistry data onto lower dimensional feature spaces. Each point in the visualization represents a single lignin structure and the color mapping denotes specific polymer properties (e.g., molecular weight, monomer count, linkages). The TMAP projection algorithm is combined with JSmol^53^ to visualize the lignin molecules in 2D and 3D representations together with their associated property data. A TMAP visualization for SG type structures is provided in the Supporting Information (Figure D). An interactive implementation of the TMAP visualization algorithm is available at https://labs.wsu.edu/pmrg/resources/lgsdataset.

## Technical Validation

A detailed comparative analysis was performed to ensure that the properties of the generated lignin structures are consistent with experimental values reported in the literature. To this end, the structure of the generated polymers (e.g., bond compositions, functional groups, and end groups) were compared with properties derived from experimental observations for MWL from Spruce and Birch wood. Results in Table 4 corroborate the agreement between generated and experimental data with degrees of polymerization of 18 and 20. Overall, the molecular weight and interunit linkage ranges for generated structures are consistent with experimental values reported in previous studies^7,54^ with the main differences due to functional group counts.

**Table 4:** Comparison of the structural compositions of generated structures and experimental data for Spruce and Birch MWL with degrees of polymerization of 20 and 18, respectively.

	Spruce – MWL		Birch – MWL	
	ref. ^5,30^	LGS Dataset	ref. ^39^	LGS Dataset
Mw (g mol^−1^)	3700	3538–3722	3294	3326–3570
Degree of polymerization	20	20	18	18
**Interunit linkages**				
β-O-4 (β-aryl ether)	12	12–14	12	10–13
β-β (Resinol)	1	1–2	3	2–3
β-5 (phenylcoumaran)	2	2–3	1	1 – 2
4-0-5 (Diaryl ether)	1	1	1	1–2
5-5 (Biphenyl) or DBDO (Dibenzodioxocin)	1	1	0	0–1
**Functional groups**				
OH-Phenolic (free)	2	2–4	4	3–7
OH-Aliphatic (primary)	11	11–14	N/A	10–13
OH-Aliphatic (secondary)	18	16–18	13	11–14
End group - Cinnamyl alcohol	2	0–1	2	0–2

**Fig. 10:**
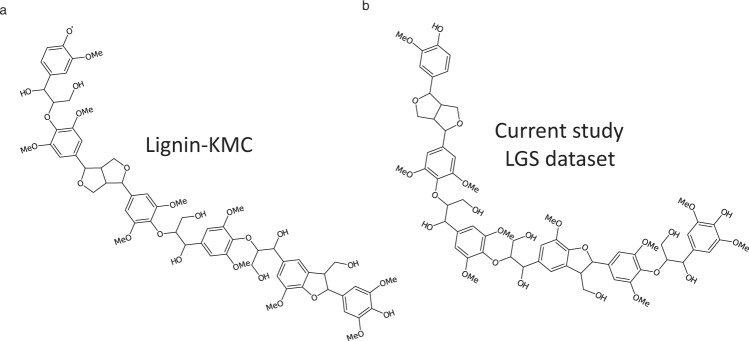
Comparison between the structures obtained with S/G ratio = 1.8 and DP = 6 using (**a**) Lignin-KMC model and (**b**) in LGS dataset.

Additional validation was performed by comparing the structures in the LGS dataset with structures generated using the Lignin-KMC model^20^. The Lignin-KMC model implements a structure generation approach based on kinetically controlled radical coupling reactions^55^. Figure 10 provides examples of lignin structures generated by each model for the same S/G ratio and degree of polymerization. Although the two lignin polymers are different, both KMC and LGS generated structures have similar features (e.g., linkage forms that includes β-aryl ether, resinol and phenyl coumaran).

**Fig. 11:**
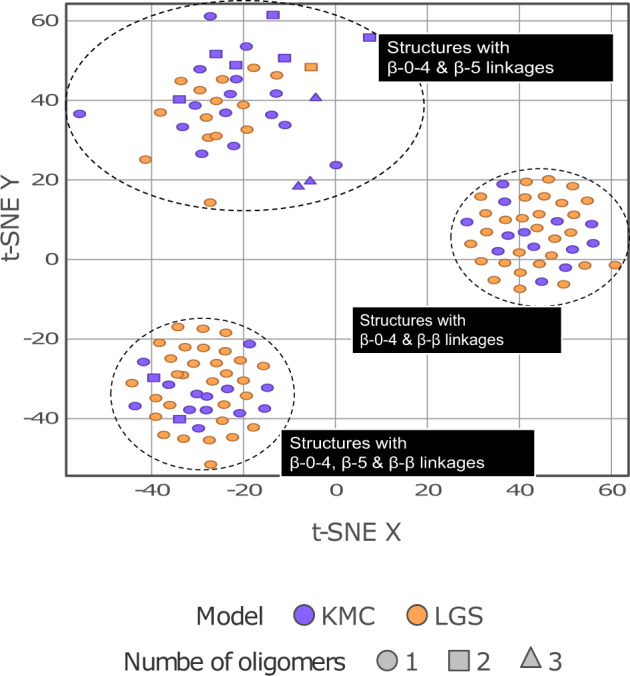
SG type structural similarities between the LGS and KMC model using t-SNE clustering on molecular structural fingerprint of structures with DP = 6.

The cheminformatics software package Datawarrior^56^ was used to inspect the differences across lignin structures with DP = 6 generated using each modeling approach. Molecular structures were encoded using binary fingerprints^57^ and projected onto a 2D space using t-distributed stochastic neighbor embedding (t-SNE)^58^. The three clusters identified in Fig. 11 group molecules with similar structural patterns. Structures in the LGS dataset overlap in each cluster with structures generated by Lignin-KMC, with small variations in the number of oligomers and cinnamyl alcohol end groups.

**Fig. 12:**
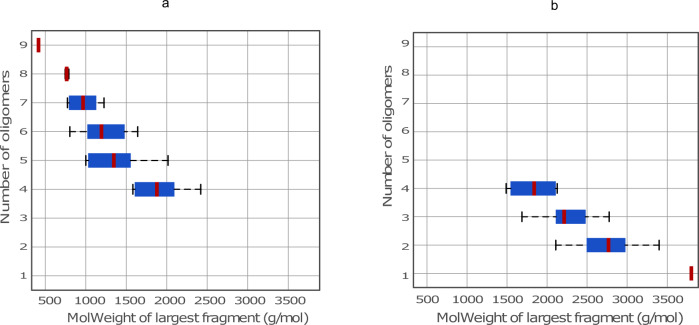
Number of oligomers vs. molecular weight of the largest oligomer for SG type structures with mean value marked in red from the LGS dataset and Lignin-KMC model for DP = 18 (**a**) Lignin-KMC model (**b**) LGS dataset.

At a higher degree of polymerization (i.e., DP = 18), the LGS model generates molecular structures with higher frequency of β-O-4 linkages that result in molecular structures with longer oligomer chains relative to those generated by the lignin-KMC model. Unlike the KMC model, the LGS approach can generate dibenzodioxocin(5-5/β-O-4/α-O-4) linkages resulting in a more diverse set of lignin structures. Figure 12 shows the difference in the molecular weight and number of oligomers for the largest lignin chain generated by each model. The LGS approach generates structures that are in good agreement with the experimental observations for MWL with larger oligomers and higher β-O-4 frequencies relative to the lignin-KMC model.

## Usage Notes

The LGS dataset constitutes a unique resource that provides primary structural data for milled wood lignin. The information included in the dataset can be used for molecular mechanics and predictive modelling studies on lignin. The dataset can also be used as a foundation to simulate lignin depolymerization into low molecular weight compounds. For instance, lignin depolymerization using deep eutectic solvents (DES) produces lower molecular weight compounds through a selective ether bond cleavage^59^ that can be simulated using the MWL structures included in the LGS dataset.

Along with MWL dataset, this study developed a computational framework to derive the structure of the lignin macromolecule by simulating longer polymer structures based on the molecular linkage patterns present on experimental data. Although spatial (i.e., 3D) arrangements of lignin macromolecules were not explored as part of this study, the current work provides a foundation for future studies and model extensions to incorporate these effects leading to a more comprehensive understanding of the molecular features of lignin.

## Supplementary information

[Supplementary Material] Molecular structural dataset of lignin macromolecule elucidating experimental structural compositions

## Data Availability

The LGS dataset and the lignin structure generator tool are available on Figshare^23^ and GitHub (https://github.com/sudhacheran/lignin-structure-generator) respectively. Moving forward, the database and accompanying tools will be periodically updated and extended. The latest version of the LGS dataset can be downloaded from: https://labs.wsu.edu/pmrg/resources/lgsdataset/.
