# Supplementary material for: Molecular structural dataset of lignin macromolecule elucidating experimental structural compositions
Source: Sci Data. 2022 Oct 22;9:647. doi: 10.1038/s41597-022-01709-4 (PMC9588021; doi:10.1038/s41597-022-01709-4)
Supplement: Supplementary file 1 — [Supplementary Material] Molecular structural dataset of lignin macromolecule elucidating experimental structural compositions [file 41597_2022_1709_MOESM1_ESM.pdf]

# Molecular structural dataset of lignin macromolecule elucidating experimental structural compositions

Sudha cheranma devi Eswaran[1,2], Senthil Subramaniam[3], Udishnu Sanyal[1,2], Robert Rallo[3], Xiao Zhang[1,2,3]

**Affiliations**

1. Bioproducts Sciences and Engineering Laboratory, Washington State University, 2710 Crimson Way, Richland WA 99354 (USA)
2. Voiland School of Chemical Engineering and Bioengineering, Washington State University (USA)
3. Pacific Northwest National Laboratory, 902 Battelle Blvd, Richland, WA 99354 (USA)

Corresponding author(s): Robert Rallo (robert.rallo@pnnl.gov), Xiao Zhang (x.zhang@wsu.edu)

# Supporting Information

**Table of Contents**

Method details 2

Lignin structure generation tool architecture and implementation 2

Data Records 4

Summary of the dataset 4

Structure Visualization in 2D and 3D representations 4

TMAP visualization of SG type structures 6

Data format specifications: 6

References 9

**Tables**

Table A: Summary of the LGS Dataset ........ 4
Table B: Example of SMILES representation for structure with degree of polymerization as 3........ 4

**Figures**

Figure A: Software architecture of the Lignin structure generator tool........ 2
Figure B: CDK integration for molecular structure generation........ 3
Figure C: Lignin model. These models, derived from 20 monomeric units, represents MWL structures of (a) softwood (predominantly G units) and (b) hardwood (ratio of S and G unit as 1.8) using LGS tool....... 5
Figure D: TMAP visualization for SG Type Structures. Color is based on free phenolic-OH group in the molecule. Clicking on individual datapoint (circle or leaf) in the tree view displays compound information detailing the structural features and link to 3D view. ........ 6
Figure E: JSON File definition for G type structures with DP as 4........ 7
Figure F: Matrices (*.csv) file definition and corresponding 2D molecular structure representation for G type structure with DP of 4........ 8
Figure G: Example MDL MOL file V3000 generated for G type structure with DP as 3........ 9

# Method details

## Lignin structure generation tool architecture and implementation

Lignin Structure (LGS) generator tool was developed as a standalone utility for computing the network of lignin molecular structure and defining a large set of lignin molecules. Tool is implemented using Core Java, major functionality includes modified version of Heap's algorithm for finding various permutations of lignin monomer, directed graph creation using combination algorithm and creation of topological matrices, integration of CDK (Chemistry Development Toolkit) for molecular structure generation and creation of SMILES notation for structures generated, storing molecular information as MDL Mol files, evaluating the structural features and storing as JSON file. Software architecture of the lignin structure generator tool is provided in Figure A. The tool can be used to generate different structural variations for a given set of experimental observations by configuring the required parameters such as monomer ratio (S, G and H), bond frequencies (β-O-4, β–β, β-5, 4-O-5, 5-5 and DBDO) in project configuration file (project-config.yaml) file. The tool can generate chemically correct and legible 2D structure diagrams of natural lignin for all wood types that includes hardwood, softwood and herbaceous.

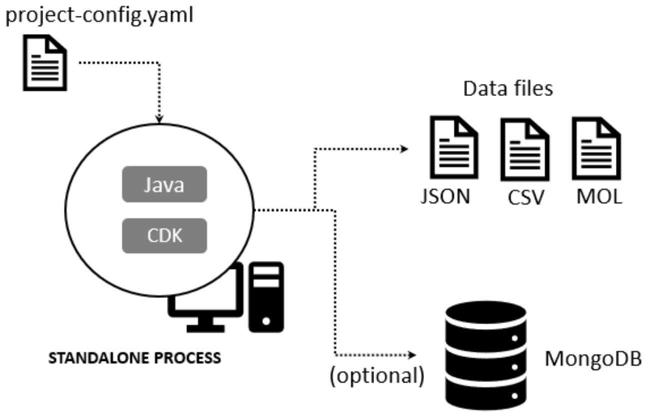


*Figure A: Software architecture of the Lignin structure generator tool*

### Molecular structure formation using CDK

CDK is a widely used open-source cheminformatics toolkit[1]. The CDK provides data structures to represent chemical concepts along with methods to manipulate such structures and perform computations on them. The monomer descriptors were initialized as IAtomContainer object from CDK and linkages between monomer units are generated using IBond object with the edge definition from the directed graphs created. Class MonolignolBase is defined an abstract class for initializing the template of monomer object containing Phenyl propane unit. Class relation diagram Figure B shows the CDK integration for molecular structure creation.

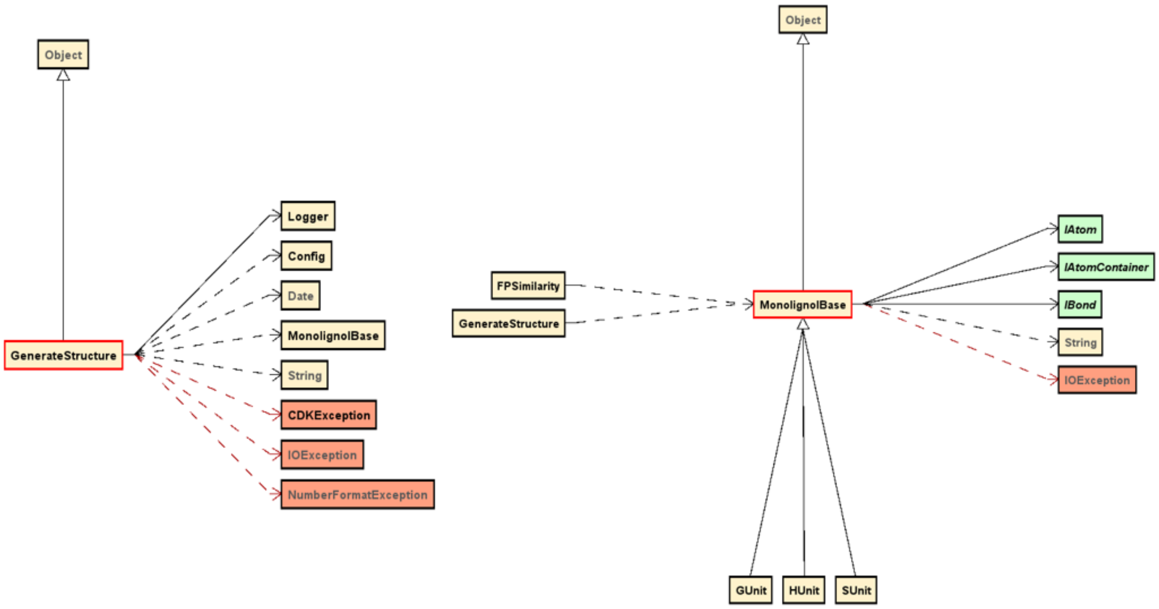


*Figure B: CDK integration for molecular structure generation*

## Implementation details

Lignin Structure (LGS) generator tool was implemented in JDK 8 using CDK 2.3, Maven 4.0.0 and MongoDB. Data analysis and charts were generated using DataWarrior v05.02.01, MongoDB and Python libraries (SciPy 1.6.2,3, Matplotlib 3.3.4,4, tMapView and RDKit 2020.09.1.0). Detailed information on the installation and execution of the Lignin structure generator tool and the source code availability can be found from the GitHub repository (https://github.com/sudhacheran/lignin-structure-generator).

# Data Records

## Summary of the dataset

Table A presents the high-level summary of the LGS dataset. The dataset contains longer polymer structures. Number of oligomers represents the range of fragmented structures present in the dataset with respect to number of monomers.

*Table A: Summary of the LGS Dataset*

| Number of monomers | G Type structures | | | SG Type Structures | | |
|---|---|---|---|---|---|---|
| | Number of structural variations | Molecular weight range (g/mol) | Number of oligomers | Number of structural variations | Molecular weight range (g/mol) | Number of oligomers |
| 3 | 4 | 504 - 520 | 1 | 6 | 560 - 576 | 1 |
| 4 | 5 | 688 - 704 | 1 | 8 | 722 - 788 | 1 - 2 |
| 5 | 10 | 873 - 889 | 1 | 62 | 957 - 973 | 1 - 2 |
| 6 | 18 | 1057 - 1073 | 1 | 131 | 1169 - 1185 | 1 - 2 |
| 7 | 24 | 1241 - 1257 | 1 | 158 | 1353 - 1369 | 1 - 2 |
| 8 | 81 | 1409 - 1425 | 1 | 219 | 1565 - 1581 | 1 - 2 |
| 9 | 123 | 1593 - 1609 | 1 | 203 | 1777 - 1793 | 1 - 2 |
| 10 | 144 | 1777 - 1793 | 1 | 929 | 1913 - 1961 | 1 - 3 |
| 11 | 411 | 1913 - 2097 | 1 - 2 | 870 | 2125 - 2173 | 1 - 3 |
| 12 | 220 | 2113 -2297 | 1 - 2 | 1081 | 2337 - 2385 | 1 - 3 |
| 13 | 529 | 2281 -2465 | 1 - 2 | 1109 | 2521 - 2569 | 1 - 3 |
| 14 | 356 | 2465 - 2650 | 1 - 2 | 1316 | 2734 - 2782 | 1 - 3 |
| 15 | 257 | 2634 - 2818 | 1 - 2 | 1323 | 2946 - 2978 | 1 - 4 |
| 16 | 296 | 2834 - 3018 | 1 - 2 | 1561 | 3130 - 3162 | 1 - 4 |
| 17 | 296 | 3002 - 3186 | 1 - 2 | 2456 | 3310 - 3358 | 1 - 4 |
| 18 | 311 | 3186 - 3370 | 1 - 2 | 5300 | 3538 - 3722 | 1 - 4 |
| 19 | 315 | 3370 - 3554 | 1 - 2 | 4720 | 3706 - 3738 | 1 - 4 |
| 20 | 263 | 3538 - 3722 | 1 - 2 | 5294 | 3902 - 4086 | 1 - 5 |
| 21 | 311 | 3722 - 3906 | 1 - 2 | 4639 | 4102 – 4134 | 1 - 4 |
| 22 | 315 | 3906 - 4090 | 1 - 2 | 5242 | 4314 - 4346 | 1 - 5 |
| 23 | 618 | 4074 - 4259 | 1 - 2 | 4830 | 4511 - 4559 | 1 - 4 |
| 24 | 685 | 4259 - 4443 | 1 - 2 | 4061 | 4679 - 4711 | 1 - 5 |
| 25 | 681 | 4443 - 4627 | 1 - 2 | 8251 | 4891 - 4923 | 1 - 5 |

## Structure Visualization in 2D and 3D representations

Lignin structures included in the LGS dataset can be visualized as 2D and 3D representations. Figure C shows 2D and 3D visualizations of G and SG type structures using CDK and Avogadro, respectively.

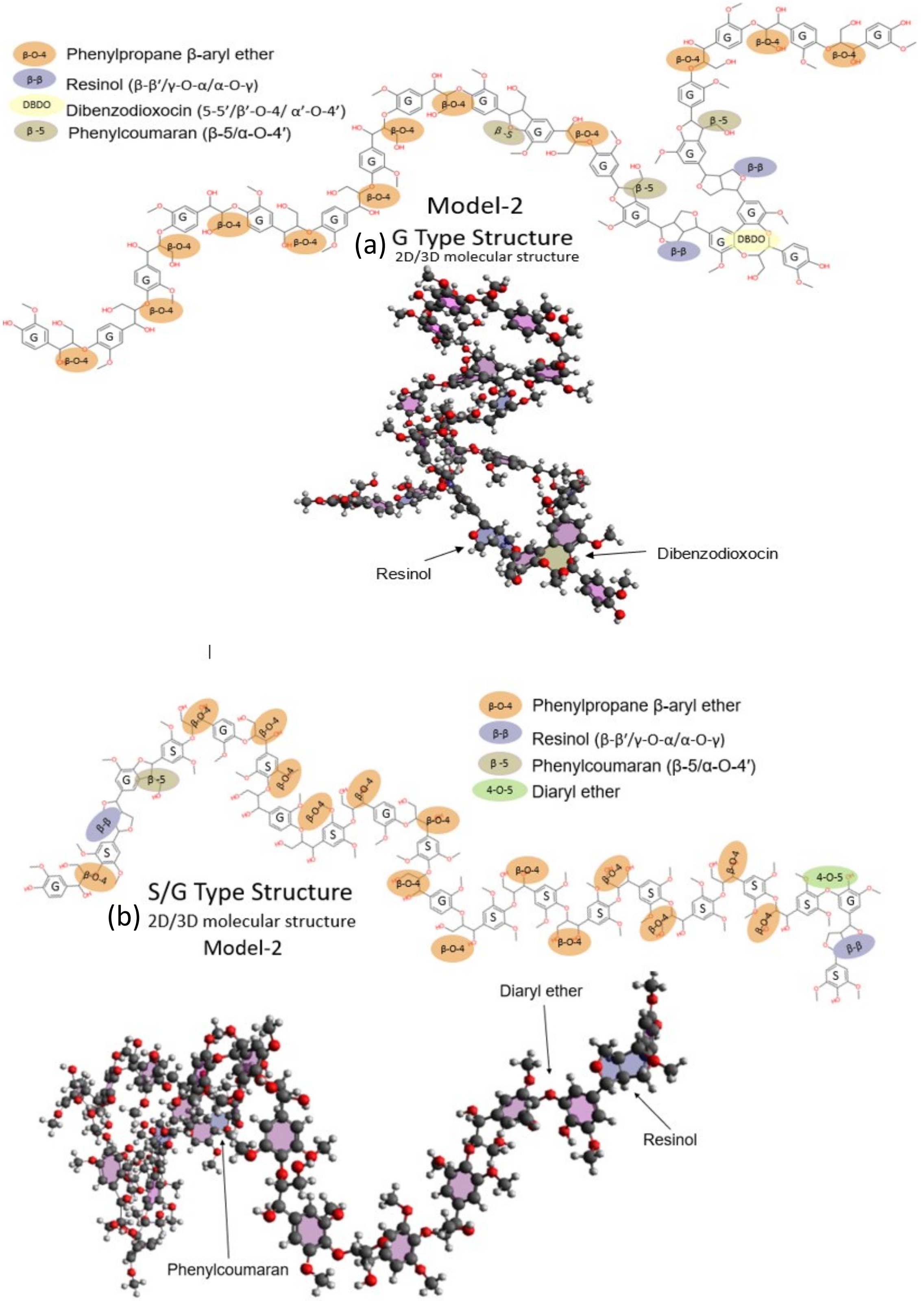


Figure C: Lignin model. These models, derived from 20 monomeric units, represents MWL structures of (a) softwood (predominantly G units) and (b) hardwood (ratio of S and G unit as 1.8) using LGS tool.

## TMAP visualization of SG type structures

Figure D shows the SG type structural dataset using Tree MAP algorithm.

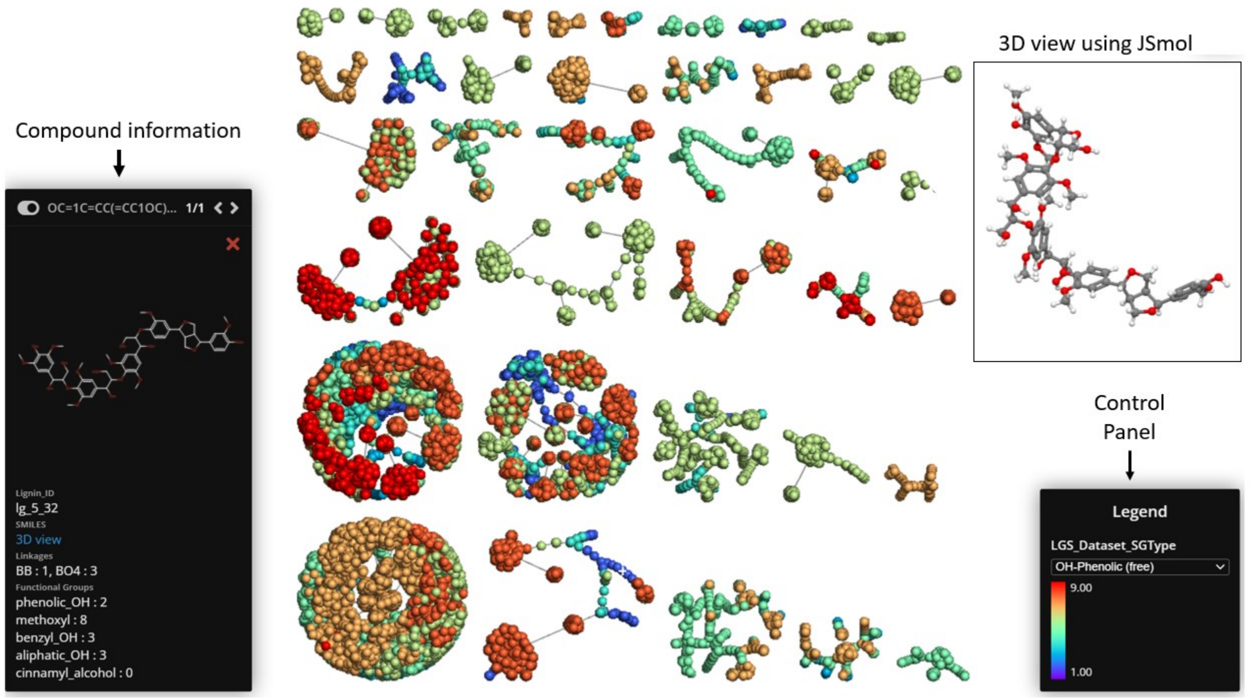


*Figure D: TMAP visualization for SG Type Structures. Color is based on free phenolic-OH group in the molecule. Clicking on individual datapoint (circle or leaf) in the tree view displays compound information detailing the structural features and link to 3D view.*

## Data format specifications:

### SMILES notation

The Simplified Molecular-Input Line-Entry System (SMILES)[2, 3] is a line notation for describing chemical structures using short ASCII strings. Lignin structures are stored as SMILES string representations of the generated molecules, as it is a key asset in cheminformatics and is becoming increasingly relevant to the general chemical community, due to the steadily growing impact of Big Data and Machine Learning. Example of the SMILES notation is shown in Table B

*Table B: Example of SMILES representation for structure with degree of polymerization as 3*

| Lignin Oligomer | Molecular structure | SMILES notation |
|---|---|---|
| S1 – βO4 – S2<br>S2 – ββ – G3 | 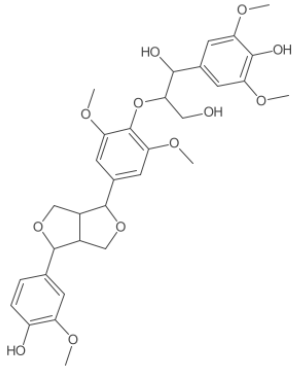 | OC=1C=CC(=CC1OC)C2OCC3C(OCC23)C4=CC(OC)=C(OC(CO)C(O)C5=CC(OC)=C(O)C(OC)=C5)C(OC)=C4 |

## JSON format

We used JSON (JavaScript Object Notation) to integrate structural features of all possible permutations for a given degree of polymerization. JSON is widely used data-interchange format. A common data format with diverse uses and stores data as key/value pairs. JSON data definition used in this study is presented in Figure E: JSON File definition for G type structures with DP as 4. The JSON datafile can be directly imported into database such as MongoDB for easy data analysis. This file provide the catalog of structural information with specific DP. Llignin id (lig_id) in the JSON object is the unique id to locate the properties of specific structure in MOL and CSV file respectively.

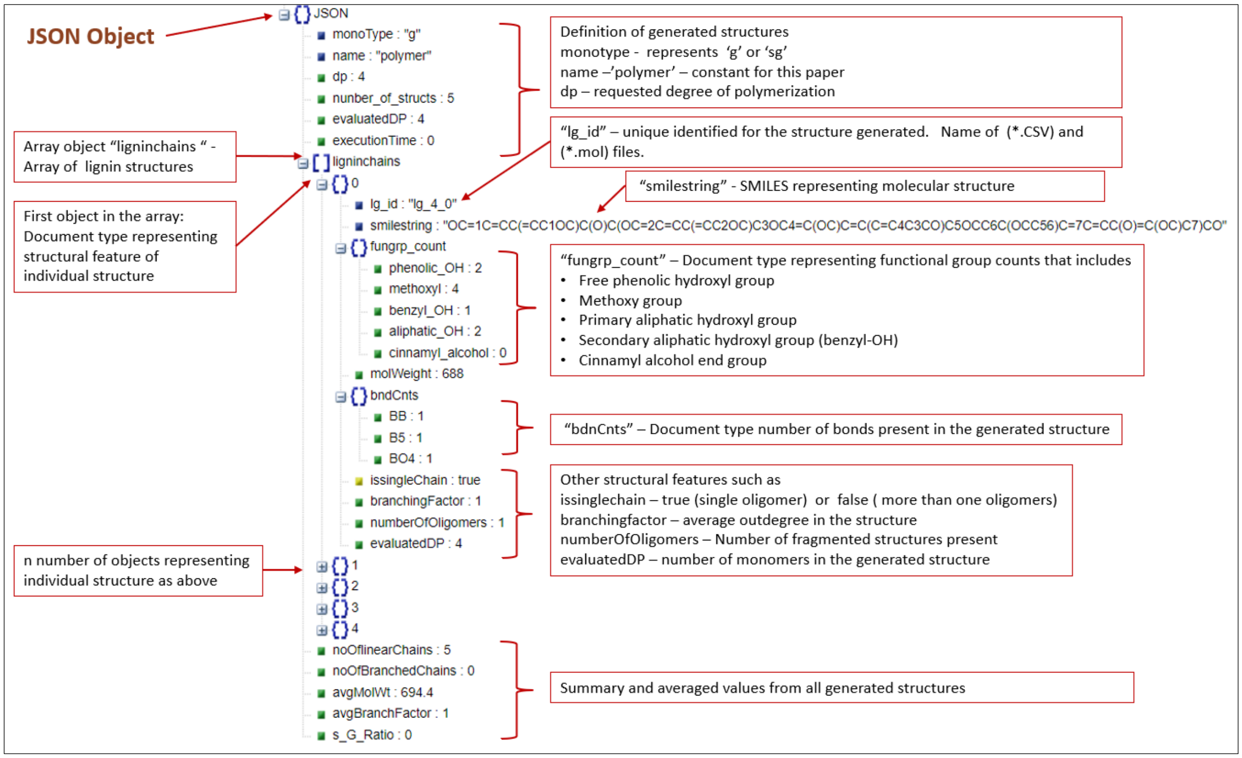

*Figure E: JSON File definition for G type structures with DP as 4*

## Matrices:

Machine readable form of molecular structure representing linkages between monomers given as adjacency and connectivity matrices. It represents the presence of linkage between the monomers (adjacency) and type of linkage between monomers (connectivity) respectively. Example of a CSV file and its fields definition is explained in Figure F.

Filename - lg_4_0.csv ← "lg_id" in JSON object

Unique id → Lignin ID

Monomers in the lignin chain → 1G, 2G, 3G, 4G

| Lignin ID | 4_0 | | | |
|---|---|---|---|---|
| Adjacency matrix | | | | |
| | 1G | 2G | 3G | 4G |
| 1G | 0 | 1 | 0 | 0 |
| 2G | 0 | 0 | 1 | 0 |
| 3G | 0 | 0 | 0 | 1 |
| 4G | 0 | 0 | 0 | 0 |
| | | | | |
| Connectivity matrix | | | | |
| | 1G | 2G | 3G | 4G |
| 1G | 0 | B | 0 | 0 |
| 2G | 4 | 0 | B | 0 |
| 3G | 0 | 5 | 0 | B |
| 4G | 0 | 0 | B | 0 |

'1' – linkage exist
'0' – no linkage

'B' – Beta bond
'4' – 4th carbon in the ring structure
'5' – 5th carbon in the ring structure

1G - βO4 - 2G
2G – β5 - 3G
3G – ββ - 4G

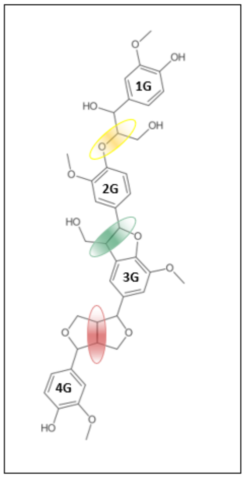


*Figure F: Matrices (*.csv) file definition and corresponding 2D molecular structure representation for G type structure with DP of 4*

## Molecular data file:

MOL files are generally classified as data files that contain molecular data information, atom, bonds, coordinates, and connectivity information in plain text format. It was developed and published by Molecular Design Limited (MDL). MDL molfile version V3000 is generated for lignin structures using CDK (Chemistry Development Toolkit). V3000[4] is used for representing proteins and polymers structures. The Avogadro software can be used in Microsoft Windows based systems, Linux and Mac OS to access and view MOL files. Example of a MOL file is provided in Figure G.

Filename: lg_3_1.mol

```
  CDK     12042113372D

  0  0  0     0  0            999 V3000
M  V30 BEGIN CTAB
M  V30 COUNTS 40 43 0 0 0
M  V30 BEGIN ATOM
M  V30 1 C 11.6 -2.59808 0 0
M  V30 2 C 13.10083 -2.5976 0 0
M  V30 3 C 13.85083 -3.89663 0 0
M  V30 4 C 13.1 -5.19615 0 0
M  V30 5 C 11.59917 -5.19663 0 0
M  V30 6 C 10.84917 -3.8976 0 0
M  V30 7 C 10.85 -1.29904 0 0
M  V30 8 C 9.35 -1.29904 0 0
M  V30 9 C 8.6 -2.59808 0 0
M  V30 10 O 9.35 -3.89711 0 0
M  V30 11 O 13.85 -6.49519 0 0
M  V30 12 C 16.10062 -5.19603 0 0
M  V30 13 O 15.35083 -3.89687 0 0
M  V30 14 C 4.1 -0 0 0
M  V30 15 C 4.85 1.3 0 0
M  V30 16 C 6.35 1.3 0 0
M  V30 17 C 7.1 -0 0 0
M  V30 18 C 6.35 -1.3 0 0
M  V30 19 C 4.85 -1.3 0 0
M  V30 20 C 2.6 0 0 0
M  V30 21 C 1.72 1.21 0 0
M  V30 22 C 2.18528 2.63601 0 0
M  V30 23 O 1.18295 3.75196 0 0
M  V30 24 O 8.6 -0 0 0
M  V30 25 C 8.60021 2.59868 0 0
M  V30 26 O 7.10021 2.59892 0 0
M  V30 27 O 11.6 -0 0 0
M  V30 28 C -2.31 0.75 0 0
M  V30 29 C -2.31 -0.75 0 0
M  V30 30 C -1.01 -1.5 0 0
M  V30 31 C 0.29 -0.75 0 0
M  V30 32 C 0.29 0.75 0 0
M  V30 33 C -1.01 1.5 0 0
M  V30 34 C -3.60892 1.50021 0 0
M  V30 35 C -4.90808 0.75042 0 0
M  V30 36 C -6.20699 1.50062 0 0
M  V30 37 O -7.50615 0.75083 0 0
M  V30 38 O 1.72 -1.21 0 0
M  V30 39 C -2.30904 -3.75 0 0
M  V30 40 O -1.01 -3 0 0
M  V30 END ATOM
M  V30 BEGIN BOND
M  V30 1 2 1 2
M  V30 2 1 2 3
M  V30 3 2 3 4
M  V30 4 1 4 5
M  V30 5 2 5 6
```

```
M  V30 5 2 5 6
M  V30 6 1 1 6
M  V30 7 1 1 7
M  V30 8 1 7 8
M  V30 9 1 8 9
M  V30 10 1 4 11
M  V30 11 1 9 10
M  V30 12 1 3 13
M  V30 13 1 13 12
M  V30 14 2 14 15
M  V30 15 1 15 16
M  V30 16 2 16 17
M  V30 17 1 17 18
M  V30 18 2 18 19
M  V30 19 1 14 19
M  V30 20 1 14 20
M  V30 21 1 20 21
M  V30 22 1 21 22
M  V30 23 1 17 24
M  V30 24 1 22 23
M  V30 25 1 16 26
M  V30 26 1 26 25
M  V30 27 1 8 24
M  V30 28 1 7 27
M  V30 29 2 28 29
M  V30 30 1 29 30
M  V30 31 2 30 31
M  V30 32 1 31 32
M  V30 33 2 32 33
M  V30 34 1 28 33
M  V30 35 1 28 34
M  V30 36 2 34 35
M  V30 37 1 35 36
M  V30 38 1 31 38
M  V30 39 1 36 37
M  V30 40 1 30 40
M  V30 41 1 40 39
M  V30 42 1 20 38
M  V30 43 1 21 32
M  V30 END BOND
M  V30 END CTAB
M  END
```

*Figure G: Example MDL MOL file V3000 generated for G type structure with DP as 3*

# References


1. Willighagen, E.L., et al., *The Chemistry Development Kit (CDK) v2.0: atom typing, depiction, molecular formulas, and substructure searching.* Journal of Cheminformatics, 2017. **9**.
2. Weininger, D., *Smiles, a Chemical Language and Information-System .1. Introduction to Methodology and Encoding Rules.* Journal of Chemical Information and Computer Sciences, 1988. **28**(1): p. 31-36.
3. Weininger, D., A. Weininger, and J.L. Weininger, *Smiles .2. Algorithm for Generation of Unique Smiles Notation.* Journal of Chemical Information and Computer Sciences, 1989. **29**(2): p. 97-101.
4. *Chemical Table files*, in *Chapter 10: The Extended Connection Table (V3000)*. 2005, Elsevier MDL.
